# Impact of short-term tinnitus treatment on cognitive function and neural synchronization

**DOI:** 10.3389/fneur.2025.1478033

**Published:** 2025-02-26

**Authors:** Ho Yun Lee, Seung-Ho Shin, Sung Wan Byun

**Affiliations:** Department of Otorhinolaryngology-Head and Neck Surgery, Ewha Womans University School of Medicine, Seoul, Republic of Korea

**Keywords:** tinnitus, treatment, cognition, neuromodulation, electroencephalogram

## Abstract

We aimed to evaluate the impact of short-term tinnitus treatment on cognitive function and identify the effects of various treatment combinations on cognitive and tinnitus outcomes. A non-randomized prospective study was conducted with 32 tinnitus patients at a tertiary university hospital between May 2022 and May 2024. Patients received treatments, including neuromodulation, diuretics, gabapentin, selective serotonin reuptake inhibitors (SSRI), anxiolytics, muscle relaxants, hearing aids, and counseling. Cognitive function and tinnitus distress were assessed using the Mini-Mental State Examination (MMSE) and Tinnitus Handicap Inventory (THI) at baseline and 1 month after treatment. Quantitative electroencephalogram (qEEG) recordings were analyzed to evaluate changes in neural synchronization using phase-locking value (PLV). Strong correlations were also observed between baseline MMSE and changes in MMSE post-treatment (r = −0.796, *p* < 0.01) and between tinnitus loudness perception and changes in MMSE (r = 0.458, *p* < 0.01). After Bonferroni correction, muscle relaxants (*p* = 0.017) and neuromodulation (*p* = 0.007) showed significant negative effects on cognitive function, while anxiolytics demonstrated a tendency for negative effects (*p* = 0.052). Additionally, neither baseline tinnitus loudness nor changes in loudness perception (ΔVAS for loudness) were significantly correlated with ΔTHI after Bonferroni correction (*p* > 0.05). qEEG analysis showed increased PLV in prefrontal-limbic and parietal-occipital connections in patients with improved THI as well as increased PLV in temporal-limbic connections in patients with improved MMSE scores, indicating enhanced neural synchronization and cognitive resource reorganization. These findings underscore the need for careful consideration of cognitive effects when selecting tinnitus treatments and highlight the importance of targeted multimodal interventions to address both tinnitus distress and cognitive function.

## 1 Introduction

Tinnitus is characterized by the perception of sound without an external source, which can disrupt cognitive functions due to associated discomfort. Its prevalence increases with age and is frequently linked to hearing loss, a known risk factor for cognitive decline (1). Auditory deafferentation resulting from hearing loss can lead to disinhibition in the auditory cortex, causing hyperactivity—a neural correlate frequently associated with tinnitus. This mechanism highlights the critical role of hearing loss in the development and persistence of tinnitus. Beyond central gain adaptation, additional mechanisms, including stochastic resonance, may contribute to tinnitus development by amplifying sub-threshold auditory signals through internally generated neural noise (2). Moreover, factors such as attentional focus, failure of sensory gating, and persistent memory traces may further reinforce tinnitus perception (3). These processes highlight the complex interplay between neural plasticity and tinnitus persistence. Several studies have reported an association between tinnitus and an increased risk of dementia and neurodegenerative diseases. A retrospective cohort study using Taiwan’s National Health Insurance data revealed that over a 10-year follow-up period, patients with tinnitus had a 1.54-fold increased risk of developing Alzheimer’s disease and a 1.56-fold increased risk of Parkinson’s disease (4). Furthermore, an increased incidence of early-onset dementia has been reported among individuals with tinnitus (5).

The link between hearing loss, cognitive decline, and dementia is well-established (1). Hearing loss, a modifiable risk factor for dementia, is known to affect cognitive domains such as executive function and memory, potentially through mechanisms like social isolation and increased cognitive load. In older adults, age-related sensory impairments combined with cognitive frailty exacerbate dementia risk, particularly when hearing loss coexists with chronic tinnitus. Interventions like hearing aids or cochlear implants may mitigate cognitive decline by reducing the sensory processing burden (1, 6, 7).

A Korean group reported that old patients with chronic bothersome tinnitus tended to have mild cognitive impairment (MCI) (8). They also found that tinnitus patients with MCI showed differences in glucose metabolism and gray matter volume compared to MCI patients without tinnitus, suggesting a distinct neural impact of tinnitus on cognitive function (36). Cognitive decline is more likely when age-related hearing loss accompanies tinnitus (7). Conversely, tinnitus has also been associated with improved cognitive performance in non-Hispanic elderly individuals with hearing loss, possibly as a compensatory mechanism where the brain reallocates resources to adapt to auditory changes, enhancing certain cognitive pathways (9, 10). Additionally, tinnitus patients often have less severe hearing loss, which may contribute to milder cognitive impairment (11, 12). Stochastic resonance may enhance auditory processing and speech perception, potentially mitigating cognitive decline. However, the cognitive impact of tinnitus varies depending on individual factors, with both protective and detrimental effects possible.

Despite extensive research, there is currently no definitive cure for tinnitus, which presents additional challenges due to the heterogeneous nature of its etiology and symptomatology. Treatment approaches are generally aimed at managing symptoms and often involve a combination of therapies to address the diverse presentations of tinnitus across patients. Various tinnitus treatments have been applied: medications, hearing aids, sound therapy, cognitive behavioral therapy (CBT), tinnitus retraining therapy (TRT), medications for accompanying symptoms, neuromodulation, and so forth (13). However, there is limited research on the impact of these treatments on tinnitus patients’ cognitive function. One study reported that bifrontal transcranial direct current modulation combined with tailor-made notched music therapy improved dichotic verbal memory, suggesting potential cognitive benefits from tinnitus treatment (14). Prolonged use of longer-acting benzodiazepines, widely prescribed for tinnitus, anxiety, and insomnia, may contribute to cognitive impairment (15).

Given the limited understanding of how tinnitus treatments affect cognitive outcomes, this study aims to assess cognitive function in patients undergoing treatment and determine whether specific treatment modalities improve cognitive changes.

## 2 Materials and methods

### 2.1 Patient and data inquiry

This non-randomized prospective study included 32 patients who visited a tertiary university hospital for tinnitus treatment between May 2022 and May 2024. Most patients were referred from primary or secondary care facilities. The institutional review board approved this study, and written informed consent was obtained from all participants (IRB number: 2021-12-017-014). Patients’ demographic and clinical characteristics, including age, gender, tinnitus duration, laterality, associated symptoms, comorbidities, and bilateral pure-tone audiometry, were documented. To evaluate global cognitive function, we used the Mini-Mental State Examination (MMSE), a validated 30-point questionnaire assessing various cognitive domains, including orientation to place and time, registration, attention and calculation, recall, language, repetition, and complex commands (16). The Korean version of MMSE used in this study has shown acceptable internal consistency with Cronbach’s alpha ranging from 0.73 to 0.78 in previous validation studies, making it a reliable tool for cognitive assessment in clinical settings (17). Based on a literature review, the MMSE and Montreal Cognitive Assessment were identified as the most widely used tools for cognitive assessment in similar studies. Given the MMSE’s robust psychometric properties, simplicity, and ability to be completed in a shorter time, it was chosen for this study. Previous studies have shown significant differences in MMSE scores between tinnitus and control groups, indicating that tinnitus may affect global cognitive function, even if MMSE scores do not directly correlate with tinnitus severity (18). Additionally, Tinnitus Handicap Inventory (THI), Visual Analog Scale (VAS) for awareness, annoyance, loudness, effect on life, Hospital Anxiety and Depression Scale (HAD-A, HAD-D), Beck Anxiety Inventory (BAI), and Brief Encounter Psychosocial Instrument (BEPSI) at baseline and 1 month after treatment.

All treatments were supervised by a single board-certified otologist (HYL) with over 18 years of clinical experience in a tertiary university hospital and specialized expertise in tinnitus management. The primary physician directly provided TRT-based counseling and medication prescriptions, while neuromodulation, hearing aids, and sound therapy were administered by a qualified audiologist under the otologist’s supervision. Comprehensive assessments were completed within 1 week of the initial visit, and patients were followed up at two-week intervals. MMSE assessments were conducted at the first visit and repeated 4 weeks after treatment to evaluate cognitive changes.

**Figure 1:**
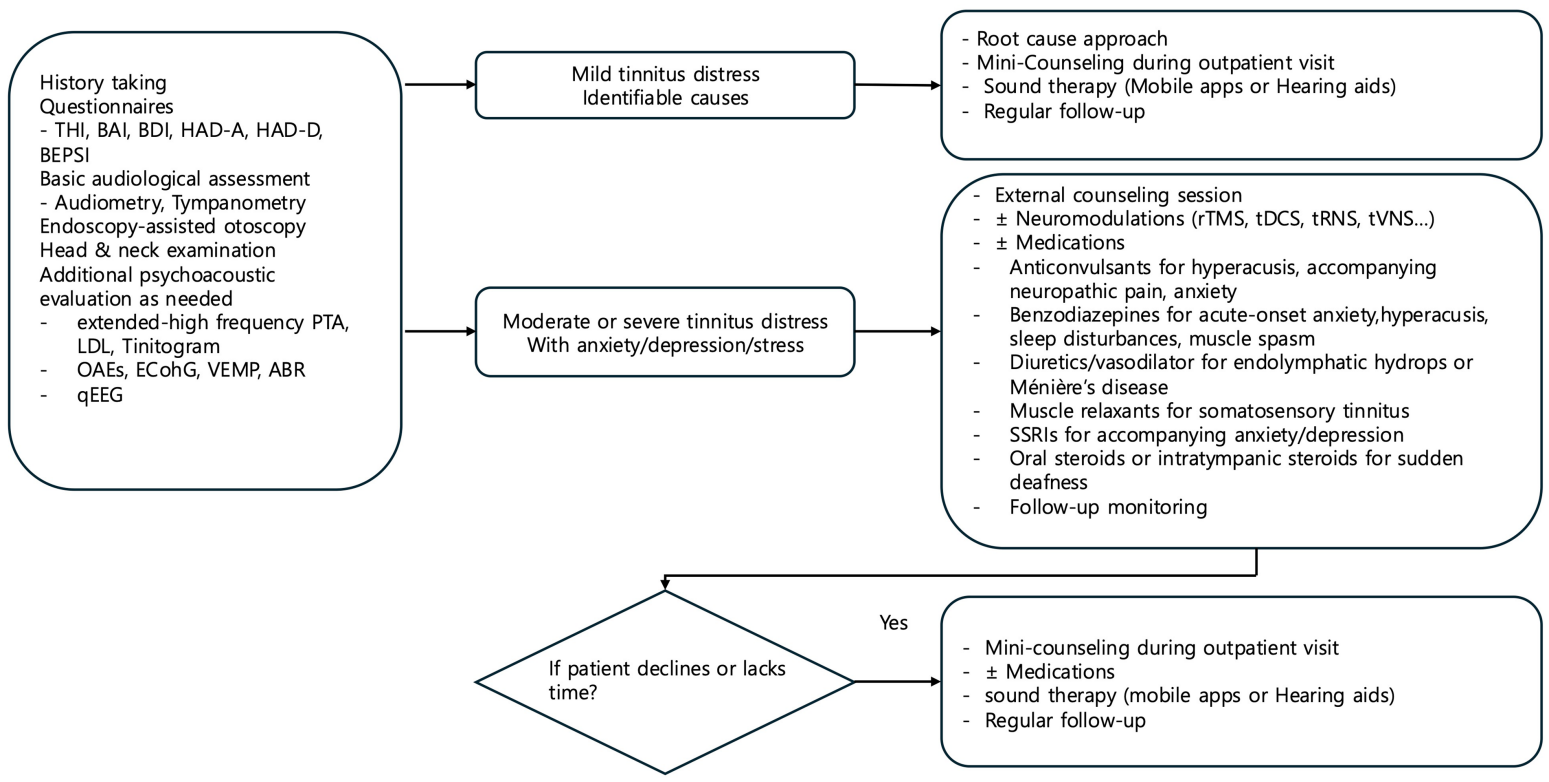
Tailored tinnitus treatment protocol from mild distress to complex cases.

The applied treatments included transcranial direct current stimulation (tDCS), diuretics, gabapentin, selective serotonin reuptake inhibitors (SSRI), anxiolytics, muscle relaxants, hearing aids, and TRT-based counseling. Based on our clinical experience in a tertiary university hospital tinnitus clinic, we developed a tailored treatment protocol for tinnitus management that addresses the diverse nature of tinnitus symptoms and patient needs (Figure 1). Our protocol begins with an initial assessment to categorize patients by the severity of their tinnitus distress and any accompanying mental health concerns. For patients with mild distress and identifiable causes, treatment focuses on addressing root causes, mini-counseling, sound therapy, and regular follow-up.

For patients with moderate to severe distress, a more intensive approach is employed, including TRT-based counseling, with neuromodulation and targeted medications as optional treatments. TRT-based counseling was provided as a single 60-min external session. TRT-based counseling includes an explanation of tinnitus as a common experience, along with discussions on its prevalence, incidence, etiologies, and possible pathomechanisms. The counseling sessions educate patients on the purposes of TRT-based counseling, including the concept of habituation and reclassification of tinnitus as a neutral sound. We also address common misconceptions about tinnitus, introduce problem-solving techniques, recommend sound therapy for tinnitus management, and encourage patients to put effort into managing their condition and to remain persistent in their treatment journey.

Neuromodulation with tDCS was applied twice weekly for up to six sessions maximally as an adjunctive treatment. The choice of neuromodulation was based on patient preference following a thorough explanation of each technique’s characteristics, benefits, and limitations. Pharmacological interventions—including SSRIs, anxiolytics, or muscle relaxants—are considered for specific symptoms based on comorbidities and examination findings, as shown in Figure 1. Regular follow-up visits were conducted biweekly over a one-month period post-treatment to monitor patient progress and make adjustments as needed, with a final assessment at the one-month mark. In cases where patients showed no symptom improvement with prescribed medications, treatment options were discussed with the patient, and modifications were made as appropriate. For patients who declined specific treatments or lacked time for more intensive therapy, we provided a simplified protocol with mini-counseling and sound therapy alone.

### 2.2 Statistical analysis

For questionnaire scores (THI, MMSE, BDI, and BAI), which typically do not follow normal distributions, values are presented as median with interquartile range [IQR], and non-parametric tests were used. Changes in these scores were analyzed using Wilcoxon signed-rank test. For correlations involving questionnaire scores, Spearman correlation analyses were conducted. Other continuous variables such as age, hearing thresholds, and VAS scores are presented as mean ± standard deviation, and parametric tests were used for these variables. Spearman correlation analyses were conducted to evaluate the associations between baseline cognitive function (measured by MMSE), hearing thresholds, THI scores, and VAS scores for tinnitus loudness and its effect on life. Although analyses were performed for bilateral hearing thresholds and all VAS parameters (awareness, annoyance, loudness, and effect on life), only significant findings are reported in the results section for clarity. To account for the increased risk of Type I errors due to multiple comparisons, Bonferroni correction was applied, and adjusted *p*-values were calculated where applicable. A stepwise linear regression analysis was performed with ΔMMSE (difference between initial and last MMSE) as the dependent variable to assess the impact of tinnitus treatments on changes in cognitive function. Independent variables included counseling, neuromodulation, diuretics, SSRI, anxiolytics, muscle relaxants, and numbers of treatment used. Treatment combinations were documented as categorical variables to evaluate the combined effect of multiple treatments on cognitive function and tinnitus distress. Separate linear regression models were constructed for ΔMMSE and ΔTHI (difference between initial and last THI) using the treatment combinations as predictors. For the stepwise linear regression analysis, *p*-values were adjusted using Bonferroni correction to account for multiple comparisons. The overall model significance was assessed using F-statistic, and individual variables’ significance was evaluated using adjusted *p*-values. Statistical analyses were performed using RStudio (version 2023.06.0 + 421 “Mountain Hydrangea”) on macOS. The software environment was Mozilla/5.0 (Macintosh; Intel Mac OS X 10_15_7) AppleWebKit/537.36 (KHTML, like Gecko) Rstudio/2023.06.0 + 421 Chrome/110.0.5481.208 Electron/23.3.0 Safari/537.36. In all analyses, a *p*-value less than 0.05 indicated statistical significance.

### 2.3 Electroencephalogram data acquisition

For quantitative electroencephalogram (qEEG), 19 channel qEEG recordings were obtained using the MINDD scan (Ybrain, Seongnam, Republic of Korea) during a resting-state period with eyes closed. Electrodes were positioned according to the international 10–20 system, with impedance below 5 kΩ and a sampling rate of 500 Hz. Each session lasted a minimum of 20 min.

Two-minute segments of EEG data free from artifacts were selected through visual inspection for analysis in this study. We utilized the advanced MATLAB toolbox, Brainstorm for EEG preprocessing, which included bandpass filtering (0.5–40 Hz) and EEG source analysis (19). We employed the sophisticated OpenMEEG as the forward model based on the Boundary Element Method (BEM) for the source modeling and utilized the ICBM152 template. Subsequently, we applied standardized low-resolution brain electromagnetic tomography (sLORETA). Functional connectivity was assessed using Phase-Locking Value (PLV) in the delta band (0.5–4 Hz), theta band (4–8 Hz), alpha (8–12 Hz), and beta (12–30 Hz), defining cortical source activity across 68 regions of interest (ROIs) based on the Desikan-Killiany atlas. PLV values between ROI pairs were used to generate connectivity matrices for each participant. Comparisons of connectivity metrics between groups were performed using nonparametric permutation t-tests with Monte Carlo *p*-values estimated from 1,000 random partitions (*p* < 0.05). Bonferroni correction was applied to determine which connectivity nodes exceeded the corrected alpha level.

## 3 Results

### 3.1 Patient characteristics

The characteristics of all patients are shown in Table 1. The average MMSE score was 28.66 ± 1.58 (range: 25–30). The treatments used for tinnitus were neuromodulation (tDCS; 93.8%, *n* = 30), anxiolytics (65.6%, *n* = 21), TRT-based counseling (43.8%, *n* = 14), diuretics (34.4%, *n* = 11), SSRI (18.8%, *n* = 6), and muscle relaxants (15.6%, *n* = 5). Additionally, three patients received gabapentin, two received beta-blockers, and one received hearing aids; these cases are described separately due to their small sample sizes.

**Table 1:** Patient characteristics.

Variables	Data
Number	32
Demographics	
Age (years)	48.03 ± 13.46
Gender (Male/Female)	24/8
Onset	42.31 ± 71.91 (Range: 1–360 month)
Hearing	
Right	19.42 ± 18.41 dB
Left	15.71 ± 15.24 dB
Tinnitus laterality (Right/Left/Bilateral/non-lateralized)	5/7/17/3
Questionnaires	
MMSE	29.00 [28.00–30.00]
THI	47.00 [34.00–72.00]
BDI	8.00 [3.00–12.00]
BAI	7.00 [1.00–12.00]
Visual analog scale	
Tinnitus Awareness	7.59 ± 2.56
Tinnitus Annoyance	7.28 ± 2.23
Tinnitus Loudness	7.02 ± 2.00
Effect on life	5.94 ± 2.27
Accompanying symptoms	
hyperacusis	6 (18.75%)
Sleep disturbance	9 (28.12%)
headache	7 (21.88%)
Neck pain	6 (18.75%)
Attention problem	10 (31.25%)
dizziness	7 (21.88%)
Aural fullness	10 (31.25%)
Accompanying diseases	
Diabetes Mellitus	2 (6.25%)
Hypertension	6 (18.75%)

MMSE, the Mini-Mental State Examination, THI, the Tinnitus Handicap inventory, BDI, the Beck Depression Inventory, BAI, the Beck Anxiety Inventory.

### 3.2 Baseline analysis and treatment outcomes

A strong negative correlation was found between baseline MMSE scores and changes in MMSE post-treatment (r = −0.796, *p* < 0.01; Table 2). Additionally, a positive correlation between VAS for loudness and ΔMMSE (r = 0.458, *p* < 0.01) suggests that patients with higher perceived tinnitus loudness experienced greater cognitive improvements following treatment.

**Table 2:** Correlation analysis of baseline MMSE and tinnitus-related variables at initial evaluation.

	Baseline MMSE	PTA (Rt)	Baseline THI	ΔMMSE	VAS for loudness	VAS for effect on life
Baseline MMSE	1	−0.339	−0.306	−0.796**	−0.398	−0.330
PTA (Rt)	−0.339	1	0.133	0.307	0.508**	0.369
Baseline THI	−0.306	0.133	1	0.214	0.462*	0.634
ΔMMSE	−0.796**	0.307	0.214	1	0.458*	0.232
VAS for loudness	−0.398	0.508**	0.462*	0.458*	1	0.589**
VAS for effect on life	−0.330	0.369	0.634**	0.232	0.589**	1

MMSE, the Mini-Mental State Examination, THI, the Tinnitus Handicap inventory, PTA, pure-tone average, VAS, Visual Analog Scale. *p* values were adjusted using Bonferroni correction. *p < 0.05, **p < 0.01.

Neither baseline VAS for loudness nor changes in tinnitus loudness (ΔVAS for loudness) showed a significant correlation with ΔTHI after Bonferroni correction (*p* > 0.05), indicating that reductions in tinnitus distress may be influenced by factors beyond perceived loudness alone. In contrast, a strong positive correlation was identified between baseline THI and VAS for effect on life (r = 0.634, *p* < 0.01), highlighting the substantial impact of tinnitus severity on patients’ perceived quality of life. After Bonferroni correction, no significant correlations were found between MMSE scores and any pure-tone averages. One month after treatment, significant changes were observed in various questionnaire scores. THI scores decreased by 35 [20.50–53.50], indicating a notable improvement in tinnitus severity (*p* < 0.001). MMSE showed a slight but statistically significant increase of 30.00 [29.00–30.00] (*p* = 0.04). Additionally, BDI scores decreased by 6.50 [4.00–9.75], and BAI scores by 7.00 [3.00–12.00], though these were not statistically significant (*p* > 0.05). VAS scores showed statistically significant improvements in awareness (1.12 ± 2.03), annoyance (1.39 ± 2.19), loudness (1.39 ± 2.15), and effect on life (1.29 ± 2.27; *p* < 0.01).

### 3.3 Change in cognitive function and tinnitus distress

The stepwise linear regression analysis identified that both muscle relaxants (*p* = 0.017) and neuromodulation (p < 0.01) had significant negative impacts on cognitive function as measured by ΔMMSE (Table 3). Anxiolytics demonstrated a tendency for negative effect (*p* = 0.052), while SSRIs and diuretics showed no significant associations (*p* > 0.05). None of these treatment modalities were associated with ΔTHI or final THI improvement (*p* > 0.05). ΔMMSE did not correlate with ΔTHI (p > 0.05), indicating that changes in cognitive function and tinnitus severity were not significantly associated in this study.

**Table 3:** Multiple linear regression analysis results for ΔMMSE.

	Estimate	Std. error	*t* value	*p* value	95% CI	
					Lower	Upper
Anxiolytics	−1.898	0.927	−2.048	0.052	−3.815	0.020
Muscle relaxant	−2.669	1.036	−2.575	0.017	−4.812	−0.525
SSRI	0.095	0.968	0.098	0.923	−1.909	2.098
Diuretics	−0.482	1.183	−0.408	0.687	−2.929	1.964
Numbers of treatment use	0.664	1.616	1.078	0.292	−0.611	1.939
Counseling	0.312	0.580	0.539	0.595	−0.887	1.511
Neuromodulation	−5.865	1.960	−2.992	0.007	−9.919	−1.810

SSRI, Selective Serotonin Reuptake Inhibitors, Adjusted *p* value: Bonferroni correction to the *p* values. Results for treatments with fewer than 4 participants (gabapentin, beta-blockers, and hearing aids) are discussed separately as case studies due to insufficient sample size for statistical analysis.

### 3.4 Case studies of less frequently used treatments

Therapeutic outcomes were analyzed separately for treatments used in fewer than four patients. Three patients received gabapentin: one received the medication with anxiolytics and muscle relaxants (ΔMMSE: +3.0, ΔTHI: −16.0), and two others received it in complex combinations with multiple medications including SSRI and neuromodulation, showing varying responses (ΔMMSE: +5.0 and 0, ΔTHI: −6.0 in both cases). Of the two patients receiving beta-blockers, one showed significant improvement while the other showed worsening when combined with different adjunct therapies (ΔMMSE: +2.0 vs. −1.0, ΔTHI: −44.0 vs. +14.0). The single patient with hearing aids showed minimal changes in both measures (ΔTHI: -8.0, ΔMMSE: 0). Due to these limited sample sizes, these observations should be considered preliminary findings requiring further investigation in larger studies.

### 3.5 Treatment combination outcomes

Table 4 summarizes the efficacy of different treatment combinations in ΔTHI and ΔMMSE. The most common treatment combinations included anxiolytics + diuretics + neuromodulation (*n* = 3) and anxiolytics + counseling + neuromodulation (*n* = 3), followed by various combinations of two or more treatments (*n* = 2). The proportion of patients who experienced a 20% or greater reduction in THI (ΔTHI ≥20%) varied across treatment combinations, ranging from 0 to 100%. A 100% THI improvement rate was observed in patients treated with muscle relaxants + counseling + diuretics + neuromodulation, diuretics + neuromodulation, anxiolytics + diuretics + beta-blockers + neuromodulation, and anxiolytics + counseling + neuromodulation + SSRI. The greatest improvement in mean ΔTHI (−66) was observed in the anxiolytics + SSRI + neuromodulation + counseling group. 50% of patients receiving anxiolytics + diuretics + neuromodulation (*n* = 2) achieved ΔTHI ≥20%. All patients treated with neuromodulation + counseling (*n* = 2), anxiolytics + diuretics + beta-blockers + neuromodulation, and anxiolytics + SSRI + diuretics + gabapentin + neuromodulation exhibited MMSE improvements (ΔMMSE ≥1%). The greatest improvement in mean ΔMMSE (+5) was observed in patients treated with anxiolytics + SSRI + diuretics + gabapentin + neuromodulation, suggesting potential cognitive benefits of this combination therapy.

**Table 4:** Combination treatment results.

Treatment combination	*n*	THI improvement rate (%)^*^ ΔTHI ≥20%	Mean ΔTHI	Cognitive improvement rate (%)^†^	Mean ΔMMSE
Anx + Cou + Diu + NM	3	33.3	−15.3	0.0	−0.3
Anx + NM + Cou	3	0.0	+17.7	66.7	0.7
Anx + SSRI + NM	2	100.0	−27.0	0.0	0.0
Anx + Diu + NM	2	50.0	−23.0	500	0.5
Anx + NM	2	0.0	−15.0	0.0	0.0
NM + Cou	2	0.0	2.0	100.0	3.0
Anx + SSRI + NM + Cou	1	100.0	−66.0	100.0	1.0
Anx + Diu + Bet + NM	1	100.0	−44.0	100.0	2.0
Mus + Diu + NM + Cou	1	100.0	−24.0	100.0	1.0
Diu + NM	1	100.0	−24.0	0.0	0.0
Anx + SSRI + Gab + Diu + NM	1	0.0	−6.0	100.0	5.0
Anx + Mus + Gab	1	0.0	−16.0	100.0	3.0
Anx + Mus + Bet + Diu + NM + Cou	1	0.0	14.0	0.0	−1
Anx + SSRI + Mus + Diu + NM + Cou	1	0.0	−6.0	0.0	−2.0
Anx + SSRI + Gab + NM + Cou	1	0.0	−6.0	0.0	0.0
Anx + Mus + NM	1	0.0	0.0	0.0	−2.0
Anx + NM + Cou + HA	1	0	−8.0	0.0	0.0

Anx, anxiolytics; NM, neuromodulation; Diu, Diuretics; Cou, Counseling; SSRI, selective serotonin reuptake inhibitors; Bet, beta-blockers; Gab, gabapentin; Mus, muscle relaxants.

* THI Improvement Rate: Percentage of patients achieving THI reduction ≥ 20 point. Negative values in ΔTHI indicate improvement.

† Cognitive Improvement Rate: Percentage of patients achieving MMSE increase ≥ 1 point. Positive values in ΔMMSE indicate improvement.

### 3.6 qEEG analysis

**Figure 2:**
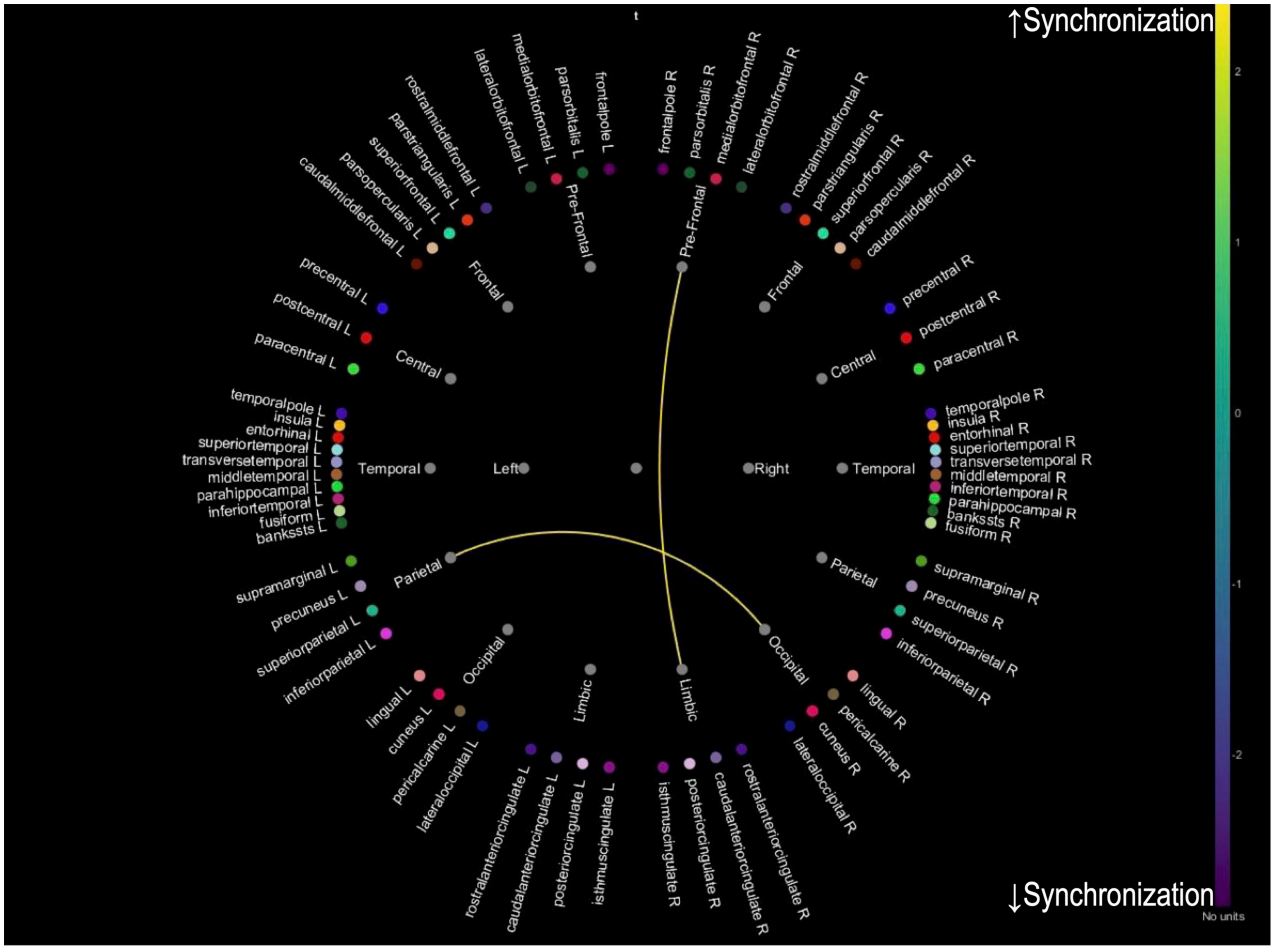
Theta band PLV connectivity in patients with improved tinnitus distress after treatment. This figure illustrates the theta band phase-locking value connectivity patterns in patients who experienced significant improvement in tinnitus symptoms following treatment. The yellow connections represent increased synchronization between prefrontal-limbic and parietal-occipital regions, associated with reduced tinnitus-related distress and enhanced emotional regulation, while blue connections indicate reduced synchronization.

**Figure 3:**
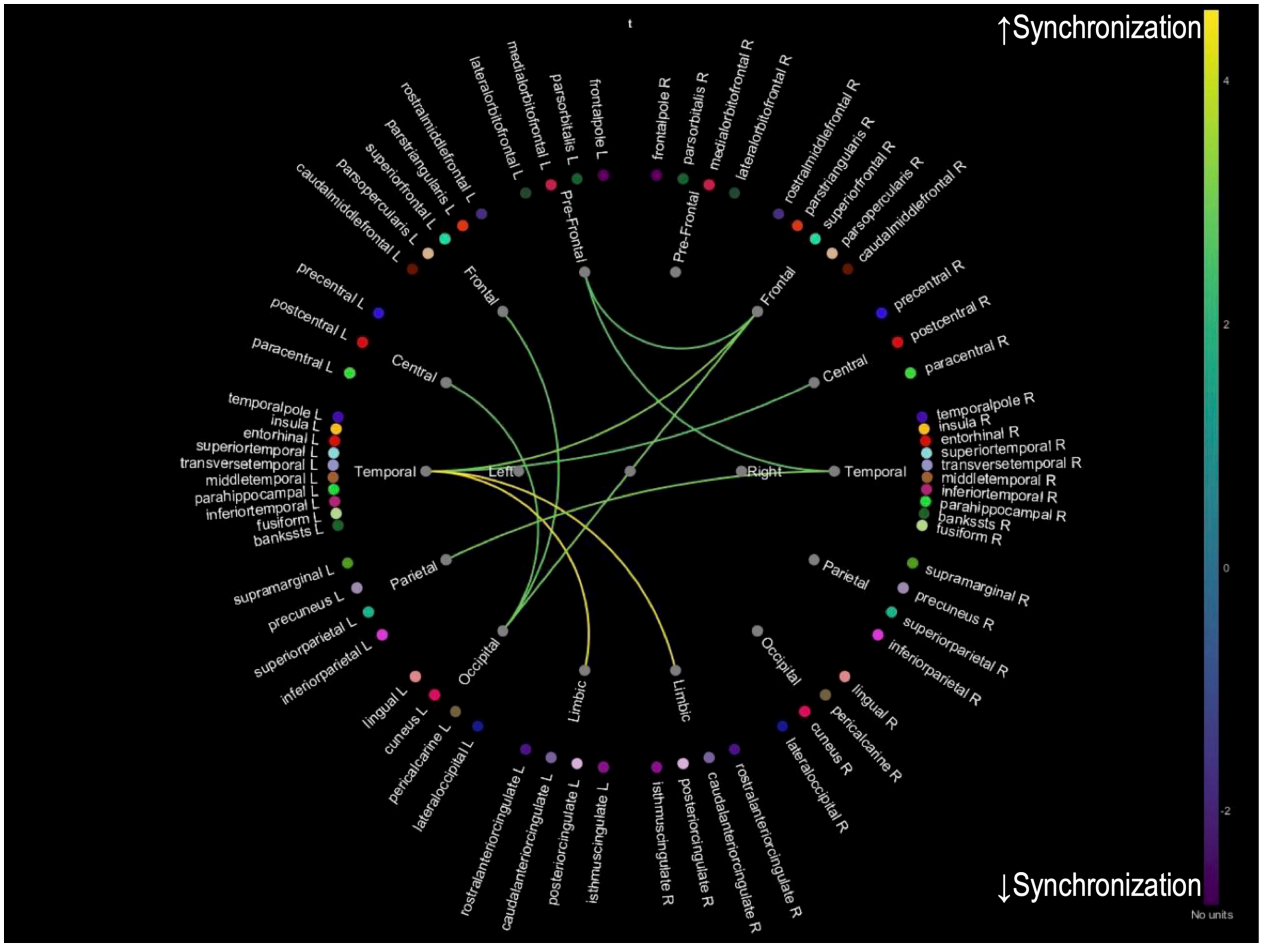
Theta band PLV connectivity in patients with improved cognition after treatment. This figure illustrates the theta band phase-locking value connectivity patterns in patients who demonstrated cognitive improvement after treatment. The yellow connections represent increased synchronization between temporal-limbic regions aligns with better memory processing and emotional regulation. Conversely, blue connections indicate reduced synchronization between parietal–temporal, frontal-occipital, and prefrontal-temporal regions, suggesting a reorganization of neural resources, indicative of improved efficiency in cognitive processing.

While we analyzed PLV changes across multiple frequency bands (delta: 0.5–4 Hz, theta: 4–8 Hz, alpha: 8–12 Hz, and beta: 12–30 Hz), only theta band analysis revealed significant distinct patterns of neural synchronization associated with improvements in tinnitus and cognitive function. Analyses of other frequency bands did not yield statistically significant results. For patients with 20 or more improved THI, an increase in PLV between prefrontal-limbic and parietal-occipital connections was observed, suggesting enhanced synchronization in these regions due to reduced tinnitus-related stress (Figure 2). For patients whose MMSE improved one or more after treatment, an increased PLV in temporal-limbic connections supports better memory and emotional regulation (Figure 3). Additionally, the latter showed decreased PLV in parietal–temporal, frontal-occipital, and prefrontal-temporal regions, indicating a potential reorganization of cognitive resources and a more efficient neural processing mechanism.

## 4 Discussion

Our findings, while limited by the small sample size (*n* = 32), provide valuable insights into the relationship between tinnitus treatment outcomes and cognitive function. Thus, the results should be interpreted considering these limitations, particularly when assessing treatment combinations and their effects.

The lack of a significant correlation between changes in THI and MMSE suggests that different mechanisms may drive improvements in tinnitus and cognitive function. The divergence in THI and MMSE changes may reflect the distinct underlying mechanisms of tinnitus-related distress and cognitive improvement, with THI more closely reflecting emotional and behavioral distress and MMSE assessing broader cognitive function. Tinnitus distress primarily influences selective cognitive domains rather than global cognitive function (20). This aligns with prior findings that suggest deficits in verbal fluency and attention-related processes while overall cognitive abilities remain preserved (21). The MMSE, while widely used, is primarily designed to screen for global cognitive impairments such as dementia and is less sensitive to detecting domain-specific changes, such as those in attention or executive function. Further studies using more sensitive instruments, such as the RBANS-H or domain-specific cognitive tasks, are needed to evaluate the nuanced cognitive impacts of tinnitus.

A significant positive correlation was observed between tinnitus loudness perception and changes in MMSE scores (r = 0.458, *p* < 0.01), suggesting that patients with greater tinnitus loudness experienced more pronounced cognitive improvements following treatment. This finding aligns with the cognitive load theory, which posits that tinnitus imposes a significant cognitive burden by continuously engaging attention resources. Effective treatment likely reduces this burden, allowing cognitive resources to be reallocated to other functions. These results are consistent with prior research showing that tinnitus-related stress predominantly affects complex cognitive functions rather than more straightforward tasks (22).

Consistent with this, our findings indicate that baseline tinnitus loudness did not significantly correlate with ΔTHI (*p* > 0.05), suggesting that absolute loudness levels at baseline are not direct predictors of tinnitus distress improvement. However, changes in loudness perception over time may still contribute to distress reduction. These findings reinforce the need to consider tinnitus loudness perception in treatment strategies, particularly for patients who subjectively experience loudness as a primary distressing factor.

Our regression analyses identified muscle relaxants (*p* = 0.017) and neuromodulation (*p* < 0.01) as having significant negative effects on cognitive function after Bonferroni correction. Anxiolytics showed a tendency toward negative effects (*p* = 0.052), while SSRIs and diuretics showed no significant associations with cognitive changes (*p* > 0.05). The negative impact of neuromodulation on cognitive function was an unexpected finding that warrants careful consideration, particularly given its frequent use in tinnitus management (93.8% of our cohort). Similarly, the negative cognitive effects observed with muscle relaxants and the tendency toward negative effects with anxiolytics suggest that these medications should be prescribed with caution, particularly in patients with cognitive concerns.

SSRIs and anxiolytics warrant careful consideration in tinnitus management. While SSRIs such as sertraline have shown efficacy in reducing tinnitus severity and loudness in previous studies, they may initially exacerbate tinnitus and cause hearing-related side effects, including auditory hallucinations and impaired auditory processing (23–25). Although our analysis did not show significant cognitive effects with SSRIs, these medications can induce various side effects, from common symptoms like nausea and insomnia to rarer but severe conditions like serotonin syndrome and suicidality, particularly in young adults aged 18–24 years (26).

Among anxiolytics, clonazepam is preferred for its longer half-life, which reduces dependency risks compared to other agents, making it a safer option for tinnitus patients requiring pharmacological management (27). While SSRIs and anxiolytics may show potential benefits for tinnitus severity reduction through management of comorbid depression and anxiety, our findings suggest they may have negative effects on cognitive function. These findings underscore the need for a balanced approach when prescribing SSRIs and anxiolytics, weighing their therapeutic benefits against potential risks.

Regarding treatment combination, notable improvements in THI scores were observed with certain treatment combinations. Particularly, the combination of anxiolytics + SSRI + neuromodulation + counseling showed the most substantial THI improvement (−66 points), suggesting a potentially synergistic effect of these modalities. The anxiolytics + beta-blockers + diuretics + neuromodulation combination also demonstrated significant improvement (−44 points). The varying responses to different treatment combinations highlight the importance of individualized treatment approaches. The combinations that achieved substantial THI improvement more than 20 points suggest that targeting multiple mechanisms may be more effective than monotherapy. However, the cognitive effects of these combinations require careful consideration, as some treatment associated with good THI outcomes showed neutral or negative effects on cognitive function (Table 4).

Notably, only one patient in our cohort received hearing aids, despite their well-documented benefits in tinnitus management. This limited use of hearing aids in our study population may reflect the general reluctance or resistance to hearing aid adoption among our patients, even though recent evidence suggests that hearing aids can be more effective than other treatments even in cases of mild hearing loss (28). This pattern highlights a potential gap between evidence-based recommendations and real-world treatment preferences, particularly in a tertiary care setting where patients might have developed specific preferences or resistance to certain treatment modalities through their previous treatment experiences. Future studies should investigate barriers to hearing aid acceptance in tinnitus patients and develop strategies to improve their adoption when appropriate, as they represent an important but potentially underutilized treatment option.

For patients whose primary symptom is tinnitus and who prefer treatments with minimal cognitive impact, counseling-based therapies such as CBT, TRT, and multisensory perceptual training (MPT) may be particularly valuable options. CBT has been the mainstream in managing tinnitus (29), and our previous reports have highlighted the importance of counseling in treating chronic tinnitus (30). MPT, which includes auditory training, visual and somatosensory integration, relaxation, and mindfulness, has also shown significant improvements in managing tinnitus symptoms (31). Interestingly, counseling was not identified as an independent prognostic factor in this study, unlike our previous research involving 151 patients. The smaller sample size (*n* = 32) may explain this discrepancy in the current study, which likely reduced statistical power and the ability to detect smaller but meaningful effects. Additionally, differences in patient characteristics, such as baseline tinnitus severity or comorbidities, may have influenced the observed outcomes. Furthermore, the current study employed multimodal treatment strategies that may have masked the independent contribution of counseling to THI improvement.

This study’s approach to tinnitus management diverges from existing guidelines by applying pharmacological and neuromodulation treatments based on individual patient history and specific clinical findings rather than routine use. The AAO guideline, for example, recommends counseling but advises against routine medical treatment without addressing individualized prescriptions based on abnormal findings (32). Similarly, the Japanese and European guidelines discourage medical treatments, citing low evidence and potential side effects, yet do not account for specific clinical abnormalities or tailored approaches (33, 34). Although recent guidelines like the German guideline recommend counseling and cognitive behavioral therapy, they do not endorse drug therapy or neuromodulation (29). In contrast, our treatment protocol applies selective treatments based on specific findings, underscoring the potential benefit of individualized care not covered in existing guidelines.

Among the pharmacological treatments, a network meta-analysis identified amitriptyline, acamprosate, gabapentin, and the combination of intra-tympanic dexamethasone injection (ITDI) plus oral melatonin as the most effective treatments for reducing tinnitus severity (35). These treatments, which have brain-acting, anti-inflammatory, and antioxidant properties, were significantly superior to placebo/control groups (35). Our treatment protocol incorporated multiple interventions to address the complex pathophysiology of tinnitus, with varying effects on cognitive outcomes (Table 4). While gabapentin showed positive effects on cognitive function, other commonly used treatments such as diuretics, anxiolytics, and muscle relaxants demonstrated adverse effects. These adverse effects may be attributed to their known side effects such as sedation and dehydration, which can impair cognitive performance. These differential effects underscore the importance of careful treatment selection based on individual patient characteristics and symptoms. Additionally, excessive focus on tinnitus can lead to cognitive impairment by depleting attentional resources.

For PLV changes in the theta band, distinct patterns of neural synchronization are associated with improvements in tinnitus and cognitive function. While preliminary analyses of alpha and beta bands were conducted, they did not yield significant results and were excluded from the final analysis. The theta band was selected for its well-documented association with cognitive processing and tinnitus-related neural activity, making it most relevant to the study’s objectives. For patients who showed an improved THI, there was an increase in PLV between prefrontal-limbic and parietal-occipital connections, suggesting enhanced synchronization in these regions due to reduced tinnitus-related stress. For those with improved MMSE scores, increased PLV was observed in temporal-limbic connections, supporting better memory and emotional regulation. Additionally, the latter exhibited decreased PLV in parietal–temporal, frontal-occipital, and prefrontal-temporal regions, indicating a potential reorganization of cognitive resources and a more efficient neural processing mechanism. These findings imply that successful treatment of tinnitus and cognitive improvements may be achieved by enhancing neural synchronization in specific brain networks. A combination of appropriate interventions may reduce tinnitus-related cognitive load and improve overall cognitive function, leading to better patient outcomes. Enhanced synchronization in key neural networks is essential for supporting both cognitive processing and auditory perception.

This study has several limitations. Our small sample size (*n* = 32) is a significant limitation, particularly for subgroup analyses comparing different treatments. While we acknowledge that *a priori* power analysis would have been beneficial for determining an adequate sample size, this was not conducted due to the exploratory nature of this clinical study. The limited sample size may affect the statistical power of our findings and increases the risk of Type II errors, particularly when examining smaller effect sizes. This limitation should be considered when interpreting our results, especially for analyses that showed non-significant findings. While adjustments for baseline anxiety, depression, and comorbidities did not significantly change the main results, these factors may still influence outcomes in specific subgroups. Larger studies with comprehensive baseline assessments and subgroup analyses are necessary to better understand the potential mediating and moderating roles of these variables. Additionally, this study was conducted at a specialized tertiary hospital in Seoul, a large metropolitan area in Korea, with expertise in tinnitus management. The hospital predominantly manages patients referred for severe or refractory tinnitus who have not improved with standard treatments elsewhere. This setting ensures a high level of expertise in managing complex cases but may limit the generalizability of findings to broader tinnitus populations. Future multicenter studies involving diverse patient groups are necessary to validate these results and expand their applicability. Furthermore, the absence of randomization may have introduced selection bias, as treatment allocation was based on clinical judgment and patient preference. This limitation underscores the need for future randomized controlled studies. The one-month follow-up period, while minimizing dropout rates, may not fully capture long-term effects of tinnitus treatments on cognitive function. Future studies should incorporate longer follow-up periods to assess the sustainability of observed improvements. The use of combination treatments poses challenges in isolating the specific efficacy of individual components, making it difficult to determine their independent contributions. Moreover, reliance on self-reported measures for tinnitus severity and cognitive function introduces potential biases. Future studies with larger, randomized cohorts and longer follow-up periods are essential to validate these findings and better understand the sustained effects of combined treatment modalities. Last, our EEG analysis was limited to two-minute segments due to our current technical expertise and established protocol. While using longer recording periods with sliding windows might provide more comprehensive data, this methodological choice was made to ensure reliable and consistent analysis within our current capabilities.

## 5 Conclusion

Our findings demonstrate that tinnitus treatment outcomes involve complex interactions between symptom relief and cognitive function, with different treatment combinations yielding distinct effects. Some combinations, such as anxiolytics + SSRI + neuromodulation + counseling, led to substantial improvements in THI, while others, like anxiolytics + SSRI + diuretics + gabapentin + neuromodulation, were associated with significant cognitive enhancement. However, the observed improvements in THI may also reflect individual differences in tinnitus distress perception rather than treatment effects alone.

qEEG analyses further revealed that these improvements corresponded with distinct patterns of neural synchronization, with enhanced prefrontal-limbic and parietal-occipital connectivity linked to tinnitus improvement, and temporal-limbic synchronization associated with cognitive enhancement. These findings suggest that separate neural mechanisms may underlie tinnitus distress reduction and cognitive function improvement.

These results emphasize the need to move beyond single-treatment approaches and instead adopt systematic, multimodal interventions that consider both tinnitus relief and cognitive effects. Given that some interventions may inadvertently impact cognitive function, it is crucial to carefully evaluate the broader implications of different treatment combinations rather than applying isolated treatments indiscriminately.

Future research should focus on validating these treatment combinations in larger populations and further refining optimized therapeutic protocols that balance tinnitus relief with cognitive preservation, ultimately leading to a more comprehensive and patient-centered approach to tinnitus care.

## Data Availability

The raw data supporting the conclusions of this article will be made available by the authors, without undue reservation.
